# Exploring Associations between Interindividual Differences in Taste Perception, Oral Microbiota Composition, and Reported Food Intake

**DOI:** 10.3390/nu11051167

**Published:** 2019-05-24

**Authors:** Camilla Cattaneo, Patrizia Riso, Monica Laureati, Giorgio Gargari, Ella Pagliarini

**Affiliations:** Department of Food, Environmental and Nutritional Sciences (DeFENS), University of Milan, 20133 Milan, Italy; camilla.cattaneo@unimi.it (C.C.); monica.laureati@unimi.it (M.L.); giorgio.gargari@unimi.it (G.G.); ella.pagliarini@unimi.it (E.P.)

**Keywords:** taste sensitivity, taste thresholds, food records, food intake, oral microbiota, eating habits

## Abstract

The role of taste perception, its relationship with oral microbiota composition, and their putative link with eating habits and food intake were the focus of the present study. A sample of 59 reportedly healthy adults (27 male, 32 female; age: 23.3 ± 2.6 years) were recruited for the study and taste thresholds for basic tastes, food intake, and oral microbiota composition were evaluated. Differences in taste perception were associated with different habitual food consumption (i.e., frequency) and actual intake. Subjects who were orally hyposensitive to salty taste reported consuming more bakery and salty baked products, saturated-fat-rich products, and soft drinks than hypersensitive subjects. Subjects hyposensitive to sweet taste reported consuming more frequently sweets and desserts than the hypersensitive group. Moreover, subjects hypersensitive to bitter taste showed higher total energy and carbohydrate intakes compared to those who perceived the solution as less bitter. Some bacterial taxa on tongue dorsum were associated with gustatory functions and with vegetable-rich (e.g., Prevotella) or protein/fat-rich diets (e.g., Clostridia). Future studies will be pivotal to confirm the hypothesis and the potential exploitation of oral microbiome as biomarker of long-term consumption of healthy or unhealthy diets.

## 1. Introduction

There are many known drivers of food choice and habits, however, taste is considered one of the main predictors [1]. It is generally assumed that humans perceive five different taste modalities: bitter, sweet, umami, sour, and salty. Each taste quality is associated with different nutritional or physiological requirements, or indicates a potential dietary risk [2]. Sweet, salty, and umami are supposed to signal the nutrient composition of foods, with sweet taste representing carbohydrates, salty taste associated with electrolytes, and umami with proteins. On the contrary, stimuli categorized as bitter and sour are associated with compounds that could be potentially harmful, and are generally regarded as innate aversions [3,4]. Taste perception varies greatly among individuals, strongly influencing food preferences and selection, and therefore nutritional status and health [5]. In particular, during the last decades, research has been focusing on bitter taste perception, and the genetic predisposition to perceive the bitter taste of 6-n-propylthiouracil (PROP) has gained considerable attention as a prototypical taste stimulus and an oral marker of food preferences and eating behavior [6]. Some additional markers include the density of fungiform papillae on the tongue tip [7] and thermal tasting [8].

Previous studies suggested a connection between individuals’ taste sensitivity and food acceptance and consumption [9,10,11]. It has been conclusively demonstrated that PROP-sensitive individuals detect more bitterness from glucosinolate-containing vegetables than non-sensitive individuals and an association between variation in bitter taste perception and food preferences has been documented [12,13,14,15]. However, the potential interaction between bitterness sensitivity and food intake has yet to be fully understood [10,16]. Furthermore, it has been reported that variation in sweet taste perception between individuals (see [17] for a review) could influence food selection and overall dietary intake. However, even if a number of previous studies [18,19,20,21,22,23] have investigated this relationship, conflicting results have been reported, probably due to differences in study participants’ characteristics (e.g., gender, ethnicity, age), in sweet taste perception assessment (e.g., psychophysical measurement, type of sweet stimuli) or in dietary intake assessment (e.g., food record, food frequency questionnaire).

The variability in response to salty stimuli has been examined for decades, but a direct genetic link to human salt taste perception has yet to be discovered [16]. The relationship between salty sensitivity and food intake has been studied much less but a connection between individuals’ salt taste sensitivity and sodium-rich foods acceptance and consumption has been suggested [18,19]. Moreover, salty sensitivity appears to be determined more by environmental factors, including exposure to NaCl and consumption of specific nutrients, than by heritability components [24,25,26,27,28].

Recently, in addition to the study of the perception of basic tastes, increasing attention has been focused on the sensitivity to fat stimulus since various evidence indicated that humans can perceive non-esterified, long-chain fatty acids in the oral cavity [29,30]. Moreover, it has been suggested that fat perception may influence the choice and consumption of some high-fat foods and, thus, possibly affects body weight [31,32]. However, additional studies are needed to confirm this assumption.

In this context, the potentiality of nongenetic factors to interact with genetic predisposition and influence food habits should be adequately considered. Recently, it has been suggested that differences in oral microbiota could be involved in the interindividual differences in taste perception. Indeed, in agreement with other reports [33,34], we previously reported a relationship between reduced taste perception and specific oral bacteria’s growth [35].

The impact of taste sensitivity and its putative influence on food intake were the focus of the present study. We explored whether variation in gustatory functions among individuals could be related to different dietary patterns and intake. Moreover, gustatory functions and dietary patterns were studied in relation to oral microbiota composition.

## 2. Material and Methods

### 2.1. Participants

Healthy, normal-weight volunteers, 18–30 years of age, were recruited from the University of Milan community through public advertisement. They received oral and written explanations of the protocol and answered questionnaires aimed at applying exclusion criteria. The following exclusion criteria were considered: (1) smokers; (2) pregnant or lactating women; (3) subjects on medication that may interfere with their ability to taste; (4) history of food allergies that may interfere with the study evaluations; (5) subjects on antibiotics two months before the study. Participants were asked to refrain from eating, drinking (except room temperature water), brushing teeth, and chewing gum for 3 h prior to testing. 

Power analysis was conducted to determine an appropriate sample size to achieve adequate power. Using data from previous studies [17,20,21] and an α of 5% and a β of 10% (90% power), it was calculated that a sample size of 51 would be required to classify at least 20% of the subjects as hypersensitive to all basic taste.

Informed, written consent was obtained from all subjects. The present study was performed according to the principles established by the Declaration of Helsinki and the protocol was approved by the Institutional Ethics Committee of the University of Milan (protocol number 16/17).

### 2.2. Gustatory Function Assessments

Seven concentrations for each taste stimulus were prepared to determine the recognition thresholds. These concentration ranges covered the published threshold values [36,37,38], and were adjusted according to preliminary tests. Concentration ranges were established such that the lowest concentration was clearly below and the highest concentration was clearly above the level at which subjects could detect or recognize the stimulus, and allowed for interindividual threshold differences. The dilution factor and the final concentration range were reported in Table 1. 

For each taste, participants received the samples at each concentration as a three-alternative forced-choice (3-AFC) ascending series, according to ISO/DIS 13301:2018 [39]. Starting from the lowest concentration, three samples (10 mL each), one containing the sample with stimulus and distilled water and two background samples (only with distilled water), were presented at each concentration. Participants were asked to take the whole 10 mL of sample into their mouth, swirl the solution around for 3 s, and expectorate. Using the forced-choice method, participants were instructed to select, or guess, the sample which was different from the other two. All samples were given a three-digit number, and the position of the samples with stimuli was randomly allocated. Participants were asked to rinse their mouth with distilled water between each concentration step.

### 2.3. Food Intake Evaluation

A Food and Beverage Frequency Questionnaire (FB-FFQ) was used to assess the consumption frequency of specific categories of foods and beverages over the previous month. The questionnaire was developed by considering a previous validated questionnaire for the Italian population [40] but specifically focusing on the main important food and beverage classes contributing to identify “consumers’ behavior according to taste sensitivity” more than actual energy and nutrient intake. In fact, the main purpose was to assess in a qualitative way the habitual intake of foods and beverages. Participants indicated their frequency of intake of 22 food categories (i.e., sweets, salty snacks, dairy products, meats, fish, fruit, vegetables) using the following frequency categories: less than once a month, 1–3 times per month, 1–4 times per week, 5–7 times per week, 2-4 times per day, and 5 or more times per day [20,41].

In addition, each participant completed a seven-day food diary to assess their food and nutrient intake. Participants were given verbal instructions and written examples on how to fill in the diary recording the type and amount of foods consumed and possibly the recipes and method of preparation. All participants were given a food record booklet. 

### 2.4. Oral Sample Collection, DNA Extraction, and Microbiota Composition Evaluation

Oral sample collection was performed as previously reported in Cattaneo et al. [35]. In brief, volunteers, sitting in front of a mirror, were asked to protrude the tongue and gently press the swab on the surface, rolling and touching edges, tip, and all defined areas of the tongue (~2/3 of tongue length) for 2 min using a sterile flocked swab (FLOQSwabsTM, COPAN S.p.A., Brescia, Italy). The swab samples were immediately placed in 750 μL of Power Bead solution provided in the DNeasy PowerLyzer PowerSoil DNA extraction kit (Qiagen, Hilden, Germany) and stored at −80 °C. For DNA extraction, samples were thawed on ice, homogenized for five minutes, and dried. Then, samples were processed using the DNA extraction kit and following manufacturer’s instructions with a minor modification (e.g., samples were incubated at 65 °C for 10 min after adding C1 solution). Bacterial cell disruption was performed mechanically using a Precellys bead beater kept in a cold room (3 cycles of 6800 rpm × 30 s; Advanced Biotech Italia s.r.l., Seveso, Italy). Quantification and verification of the 260/280 ratio of the extracted DNA was carried out with a Take3 Micro-Volume plate in a Gen5 microplate reader (BioTek Instrument Inc., Winooski, VT, USA). Finally, DNA samples were stored at −80 °C. The bacterial taxonomic composition of oral swabs was assessed in a previous study by 16S rRNA gene profiling using Illumina HiSeq technology [35]. Analysis and taxonomic assignment of sequencing reads were also performed by means of the bioinformatic pipeline Quantitative Insights Into Microbial Ecology (QIIME) version 1.9.0 with the GreenGenes database (version 13_5). Metadata were deposited in the European Nucleotide Archive (ENA) of the European Bioinformatics Institute under accession code PRJEB28769.

### 2.5. Data Analysis

The matrix of the correct and incorrect answers produced separately by each judge was used to calculate the individual thresholds. The individual’s Best Estimate Threshold (BET) for each sensory stimulus was calculated as the geometric mean of the highest concentration missed and the next highest concentration that was correctly recognized (ISO/DIS 13301:2018) [39]. 

Participants were divided according to their taste sensitivity into three groups, using basic taste thresholds as a grouping variable. Participants were defined as hypersensitive if they presented threshold values in the lower percentile (25th percentile): Salty ≤ 0.088 g/L; Sweet ≤ 0.639 g/L; Bitter ≤ 0.0164 g/L; Sour ≤ 0.0316 g/L, and as hyposensitive if they presented threshold values in the higher percentile (75th percentile): Salty ≥ 0.0353 g/L; Sweet ≥ 4.040 g/L; Bitter ≥ 0.1385 g/L; Sour ≥ 0.1095 g/L. The remaining subjects were considered as medium sensitive.

Food record data were used to estimate total energy and macronutrient intake by using the software MètaDieta developed using Italian food composition tables (METEDA srl, Italy). 

The FB-FFQ data registered for the 22 food item categories were converted to daily frequency equivalents (DFE) calculated by allocating proportional values to the original frequency categories with reference to a base value of 1.0, equivalent to once a day [20,41]. The scores were calculated as reported in Table 2. 

Pearson’s coefficients correlations were conducted to analyze the relationship between the gustatory functions.

Mixed ANOVAs were carried out considering “Taste sensitivity” (hyper, medium, and hypo) to basic tastes, “gender” (female, F and male; M) and their interaction as fixed factors and dietary intake, total energy and macronutrient intake as dependent variables, followed by pairwise comparisons using the Bonferroni test adjusted for multiple comparisons (*p* < 0.05). Participants were added as random factor in all the analyses. These statistical analyses were performed using IBM SPSS Statistics for Windows, Version 25.0 (IBM Corp., Armonk, NY, USA). 

Correlation analyses between the tongue microbial ecology data, gustatory functions, and dietary intake, total energy and macronutrient intake were performed using the Kendall and Spearman formulas as predictors and dependent variables. Significance was set at *p* ≤ 0.05 (α = 5%); significance in the range 0.05 < *p* < 0.10 was accepted as a trend.

## 3. Results

### 3.1. Participant Characteristics

Fifty-nine volunteers (27 males, 32 females) were recruited for this study. The characteristics of all participants are detailed in Table 3.

### 3.2. Association among Gustatory Functions and Their Relationship with Food Intake

Significant correlations were found between tastes that share many common features in the transduction mechanisms. In particular, the bitter threshold showed a significant correlation with the sweet threshold (*r* = 0.34, *p* < 0.01), while the sour threshold had significant correlations with the recognition thresholds of salty (*r* = 0.34, *p* < 0.01). Moreover, a significant correlation was found between sour and bitter thresholds (*r* = 0.31, *p* < 0.05). 

As previously described in the “Material and Methods” section, basic taste thresholds were used as grouping variables and respondents were divided into three groups according to their sensitivity (hypersensitive, medium sensitive, hyposensitive). Among the whole samples, no more than 14 subjects switched from hypo- to hypersensitive within the different stimuli. For salty sensitivity, the group with low sensitivity corresponded to 27.1% of the total sample (10 M, 6 F), the medium sensitive group accounted for 27.1% (7 M, 9 F) and the group with high sensitivity corresponded to 45.8% (10 M, 17 F) of the total sample. For sweet sensitivity, the group with high sensitivity corresponded to 28.8% (9 M, 8 F) of the total sample, while the medium and hyposensitive groups accounted for 22.1% (6 M, 7 F) and 49.1% (12 M, 17 F) of the total sample, respectively. For bitter sensitivity, the hypersensitive group corresponded to 40.7% (15 M, 9 F) of the total sample, while the medium and hyposensitive groups accounted for 18.6% (4 M, 7 F) and 40.7% (8 M, 16 F) of the total sample, respectively. For sour sensitivity, the hypersensitive group corresponded to 22.0% (5 M, 8 F) of the total sample, while the medium and hyposensitive groups accounted for 44.1% (10 M, 17 F) and 33.9% (12 M, 8 F) of the total sample, respectively. 

#### 3.2.1. Salty Sensitivity

The elaboration of the results on potential impact of “*Salty sensitivity*” on food and beverage consumption frequency is reported in Table 4. 

Consumption frequency of bakery and salty baked products, legumes, fats, and soft drinks seemed to be associated with “*Salty sensitivity*”. Post hoc comparisons showed that, in general, hyposensitive subjects consumed these products significantly more than did medium and hypersensitive subjects. The main factor “*gender*” was significant for various food categories. In all cases, females have been found to consume significantly less salty baked products (F_(1,53)_ = 8.46, *p* < 0.01), cured meats (F_(1,53)_ = 11.25, *p* < 0.001), and soft drinks (F_(1,53)_ = 10.19, *p* < 0.01), but more fish (F_(1,53)_ = 9.88, *p* < 0.01), fruit (F_(1,53)_ = 8.15, *p* < 0.01), and nuts (F_(1,53)_ = 7.02, *p* < 0.05) than males. The “*Salty sensitivity*” × “*gender*” interaction was significant only in a few cases (cereal and cereal-derived products: F_(2,53)_ = 5.52, *p* < 0.01; cured meats: F_(2,53)_ = 3.62, *p* < 0.05; nuts: F_(2,53)_ = 3.27, *p* < 0.05; soft drinks: F_(2,53)_ = 5.06, *p* < 0.01). 

When food record data were considered, a significant association with “*Salty sensitivity*” was found (F_(2,53)_ = 3.52, *p* < 0.05) on fat (as% energy intake), with hyposensitive subjects showing a higher intake compared to medium and hypersensitive subjects. A significant “*gender*” association was found (F_(2,53)_ = 5.76, *p* < 0.05), underlying a higher fat intake in female subjects with respect to males.

#### 3.2.2. Sweet Sensitivity

The elaboration of the results on potential impact of “*Sweet sensitivity*” on food and beverage consumption frequency is reported in Table 4. 

Consumption frequency of legumes and sweets and desserts seemed to be associated with “*Sweet sensitivity*”. Post hoc comparisons showed that, in general, hypersensitive subjects consumed these products significantly less than did medium and hyposensitive subjects. The main factor “*gender*” was significant for various food categories. In all cases, females reported consuming significantly less salty baked products (F_(1,53)_ = 14.29, *p* < 0.001), cured meats (F_(1,53)_ = 5.89, *p* < 0.05), sweets and desserts (F_(1,53)_ = 4.06, *p* < 0.05), alcoholic beverages (F_(1,53)_ = 5.19, *p* < 0.05), and soft drinks (F_(1,53)_ = 5.16, *p* < 0.05), but more fish (F_(1,53)_ = 5.70, *p* < 0.05) than males. The “*Sweet sensitivity*” × “*gender*” interaction was significant only in a few cases (dairy products: F_(2,53)_ = 3.48, *p* < 0.05; candies and gums: F_(2,53)_ = 3.17, *p* < 0.05).

When food record data were considered, significant differences were found between female and male subjects on total energy and carbohydrates and fat consumptions. Energy (kcal) (F_(2,53)_ = 4.71, *p* < 0.05) and carbohydrate intakes (g) (F_(2,53)_ = 5.70, *p* < 0.05) were significantly lower in female subjects compared to male subjects. By contrast, fat intake (as% energy intake) (F_(2,53)_ = 8.60, *p* < 0.01) was significantly higher in females than males.

#### 3.2.3. Bitter Sensitivity

The elaboration of the results on potential impact of “Bitter sensitivity” on food and beverage consumption frequency is reported in Table 5.

“*Bitter sensitivity*” had a significant association with consumption frequency of oils (F_(2,53)_ = 5.41, *p* < 0.01). Post hoc comparisons showed that hyposensitive subjects consumed these products significantly more than did medium and hypersensitive subjects. The main factor “*gender*” was significant for some food categories. In all cases, females reported consuming significantly less salty baked products (F_(1,53)_ = 6.63, *p* < 0.05) and cured meats (F_(1,53)_ = 8.47, *p* < 0.01), but more fish (F_(1,53)_ = 8.79, *p* < 0.01), fruit (F_(1,53)_ = 4.87, *p* < 0.05), and nuts (F_(1,53)_ = 4.02, *p* < 0.05) than males. The “*Bitter sensitivity*” × “*gender*” interaction was significant only in a few cases (oils: F_(2,53)_ = 5.32, *p* < 0.01; salty snacks: F_(2,53)_ = 3.54, *p* < 0.05).

When food record data were considered, a significant association with “*Bitter sensitivity*” was found on energy (Kcal) (F_(2,53)_ = 3.30, *p* < 0.05) and carbohydrates (g) (F_(2,53)_ = 3.59, *p* < 0.05) intakes, with hypersensitive subjects showing higher intakes compared to medium and hyposensitive subjects. A significant “*gender*” association was found with carbohydrates (F_(2,53)_ = 6.97, *p* < 0.01) and fat intakes (as% energy intake) (F_(2,53)_ = 11.77, *p* < 0.001), underlying a lower carbohydrates but a higher fat intake in females than males.

#### 3.2.4. Sour Sensitivity

The elaboration of the results on potential impact of “Sour sensitivity” on Food and Beverage consumption frequency is reported in Table 5. 

“*Sour sensitivity*” had a significant association only with consumption frequency of fish (F_(2,53)_ = 6.14, *p* < 0.01). Post hoc comparisons showed that subjects characterized by medium sensitivity to sour consumed fish significantly less than did hypo- and hypersensitive subjects. The main factor “*gender*” was significant for some food categories. In all cases, females reported consuming significantly less salty baked products (F_(1,53)_ = 8.21, *p* < 0.01), cured meats (F_(1,53)_ = 6.47, *p* < 0.05), and soft drinks (F_(1,53)_ = 5.29, *p* < 0.05), but more fish (F_(1,53)_ = 10.42, *p* < 0.01) and nuts (F_(1,53)_ = 4.04, *p* < 0.05) than males. The “*Sour sensitivity*” × “*gender*” interaction was significant only in one category (salty snacks: F_(2,53)_ = 3.54, *p* < 0.05).

When food record data were considered, significant differences were found between female and male subjects for carbohydrates (g) (F_(2,53)_ = 3.86, *p* < 0.05) and fat consumptions (as% energy intake) (F_(2,53)_ = 9.12, *p* < 0.01). Carbohydrates intake (g) was significantly lower in female subjects compared to male subjects. By contrast, fat intake (as% energy intake) (F_(2,53)_ = 8.60, *p* < 0.01) was significantly higher in females than males.

### 3.3. Correlation between Tongue Dorsum Microbiota, Gustatory Functions, and Dietary Intake

To infer potential links between bacteria on tongue dorsum, gustatory functions, and dietary intake, we performed correlation analyses between taste thresholds, total energy, and macronutrient intake and the DADA2/SILVA/speciateIT-determined bacterial relative abundances. 

**Figure 1:**
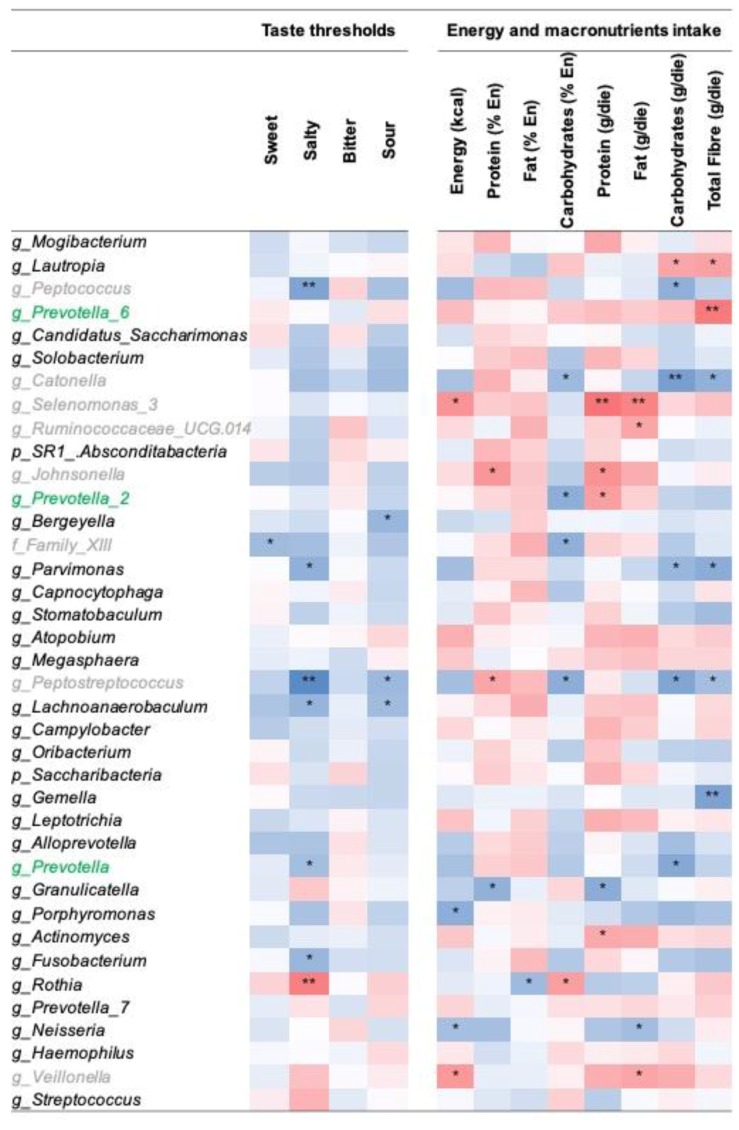
Correlations between the relative abundance of bacterial taxa on tongue dorsum and taste thresholds for the four basic tastes (left side) and nutritional variables (right side). The heatmap represents the Spearman’s correlation R-values. Asterisks relate to the Kendall rank correlation *p* values: * *p* < 0.05; ** *p* < 0.01. *Prevotella* genera and *Clostridia* class, which showed significant correlation with energy and macronutrients intake, are highlighted in green and in grey colors, respectively.

Several bacterial taxa abundances were correlated with taste thresholds. In particular, one taxon negatively correlated with sweet, three with sour, and six with salty thresholds. In summary, bacterial taxa abundances increase in subjects characterized by lower taste thresholds. On the contrary, the genus *Rothia* was the only taxon positively associated with taste thresholds, specifically salty. No taxon was correlated with the bitter threshold (Figure 1 – left side). 

Finally, we performed correlation analyses between the oral microbiota and dietary intake. We found that energy and macronutrient intake were significantly correlated with several bacteria taxa (Figure 1 – right side). Notably, we found that several taxa in *Clostridia* class (e.g., genera *Selenomonas, Ruminococcaceae, Johnsonella*, and *Veilonella*) were positively correlated with total energy, protein, and fat intake and negatively correlated with carbohydrates and total fiber intake (e.g., genera *Catonella* and *Peptostreptococcus*). Contrarily, *Prevotella* genus was positively correlated with total fiber intake (Figure 1 – right side). Overall, these results seem to indicate that some microbial taxa are positively associated with vegetable-rich (*Prevotella* genus) or protein/fat-rich diets (*Clostridia* class).

## 4. Discussion 

The present study evaluated interindividual differences in recognition thresholds for basic tastes and examined to what extent these variations in gustatory functions among individuals could be related to food intake in a sample of reportedly healthy adult women and men. Additionally, these variables were further evaluated in relation to individual oral microbiota composition. 

The present study shows that recognition thresholds for the basic tastes were associated with each other, albeit in different ways. Indeed, significant correlations were found between tastes that share many common features in the transduction mechanisms. The perception of both sweet and bitter tastes is mediated via G-coupled protein receptors, encoded by TAS1R and TAS2R taste receptor gene families, while salty and sour tastes are transduced via ion channels [42]. Thus, these findings seem to confirm the presence of the well-known dichotomy in taste coding for perception of pleasant (e.g., sweet and savory compounds) vs noxious stimuli (e.g., sour and bitter tastants) [43]. 

The results of the present study showed that interindividual differences in taste perception may influence habitual food consumption and intake. This assumption supports various observations that taste sensitivity may play an important role in dietary habits and body energy balance [37,44,45,46]. Indeed, it has been suggested that subjects characterized by a reduced or distorted taste sensitivity could increase the willingness to ingest foods that involve greater stimulation of the taste and oral somatosensory system (e.g., high-energy dense foods rich in sugars and fats), leading to unhealthy food choices, and thus pathogenesis of weight excess.

As far as salt intake is regarded, a few studies [47,48,49] examined the association between salt hedonics and sodium intake, but the relationship between salty sensitivity and food intake has been poorly investigated. Kim and Lee [25] reported an association between individuals’ salty taste sensitivity and sodium-rich fast foods acceptance and consumption in a sample of Korean adolescents. Moreover, Hayes and colleagues [47] reported that variation in salt perception was associated with differences in preferences to high-sodium foods and, indirectly, to sodium intake. Accordingly, in the present study, subjects who were orally hypersensitive to sodium chloride solution reported consuming less bakery and salty baked products than those who were defined as hyposensitive. Moreover, hyposensitivity to salty taste seems to increase consumption of less healthy foods, like saturated-fat-rich products and soft drinks. This assumption is supported by food record data, in which fat intake (expressed as a percentage of total energy intake) was found to be higher in the hyposensitive group. 

Previous studies have failed to find associations between sweet taste and diet parameters [21,23]. Contrarily, the present data from the FB-FQ and sweet sensitivity suggests that participants who have a higher threshold for sweet taste (hyposensitive) reported consuming more frequently sweets and desserts than the hypersensitive group. This is supported by findings of recent studies, where positive relationships were found between reduced perceived intensity and increased desire for higher energy providing taste stimuli [50]. Accordingly, Jayasinghe and colleagues [20] showed that participants who perceived as sweeter the highest glucose concentrations are reported to be more sensitive to sweet taste and had a lower consumption frequency of sweet foods compared to those who perceived the solution proposed as less sweet. 

Interestingly, this study did not find any relationship between sweet taste sensitivity and energy or macronutrient intakes expressed as a percentage of total energy or grams. This result was in contrast with previous findings suggesting an inverse correlation between glucose taste perception and total energy and carbohydrate intakes [20]. However, as recently discussed by Webb and colleagues [38] it is necessary to use a combination of sweet taste measurements (e.g., glucose, sucrose, and fructose) to better characterize the overall perception and the relationships between sweet taste perception and food intake.

Nevertheless, we observed a relationship between bitter taste sensitivity and total energy and carbohydrates intakes. Participants who were orally hypersensitive to caffeine solutions showed higher total energy and carbohydrate intakes compared to those who perceived the solution as less bitter, suggesting a potential shift towards less healthy dietary patterns in the hypersensitive group of subjects. These results seem to support the hypothesis that higher taste sensitivity to bitter compounds can elicit rejection responses in subjects leading to a reduced selection and intake of some vegetable foods in favour of high-energy-dense foods [51]. 

Regarding the relationship between sour taste sensitivity and food consumption frequency, our results failed to underline any significant association. It is important to note that, in literature, the attention has been mainly focused on individual variation in sour taste perception and preferences for sour foods [52], suggesting that low preference for sour foods could eventually lead to limited choices or inadequate intake of fruit and berries. However, even if these results demonstrated a genetic contribution to preference for sour foods, the authors underlined that sour taste perception and related preferences for sour foods are mediated by both genetic and environmental factors (e.g., food habits of the family). Thus, the potential relationship among sour taste perception and subsequent food choices and intake remains to be explored [16].

As expected, gender-related differences in food consumption frequency and intake were found, confirming previous studies in which differences in the nutritional quality of the diet of men and women were highlighted [53,54,55,56]. Indeed, men reported significantly more frequent consumption of salty baked products, cured meats, sweets and desserts, alcoholic beverages, and soft drinks than did women. On the other hand, women were more likely to consume fish, fruit and nuts. Macronutrients intake, in terms of percentage of total energy intake, differed between female and male subjects. Clearly, as expected, men consumed higher total energy and carbohydrates compared to women.

In order to provide further insights into the complexities of human eating behavior, the present study focused the attention on less investigated nongenetic factors potentially influencing food preference and habits. In particular, we considered the oral microbiota composition since, recently, a relationship between reduced taste perception and specific oral bacteria’s growth has been reported [35], in agreement with previous findings [33,34]. Solemdal and colleagues [34] investigated variables related to taste ability and oral health in acutely hospitalized elderly, showing that taste perception, particularly for sour taste, was reduced in acutely hospitalized elderly with high lactobacilli growth. Besnard and colleagues [33] tested the hypothesis that obese and normal-weight adults could be characterized by an impaired fat taste perception, which could be also linked to a change in the microbial composition. This study showed no difference in the fat taste perception and composition of oral microbiota between normal-weight and obese subjects. Otherwise, specific bacterial composition was found in lipid non-tasters, irrespectively of nutritional status. Moreover, in our previous study, we found that subjects who were characterized by a greater responsiveness to PROP presented differences in the relative abundance of some taxa compared to subjects who were less responsive to the PROP compound. In particular, five bacterial genera, including the Gram-positive genera *Actinomyces*, *Oribacterium*, *Solobacterium*, and *Catonella*, and the Gram-negative *Campylobacter*, were overrepresented in the most responsiveness group.

In the present study, interesting further correlations between the relative abundance of bacterial taxa on tongue dorsum and gustatory functions were found. The present results showed that a number of taxa were inversely correlated with salt and sour thresholds, showing that a great salty and sour sensitivity may be linked to specific taxa, mainly attributed to *Clostridiales* and *Bacteroidales* order. Given the diversity of genera and species within the oral microbiome [57], it is overall difficult to propose systematic explanations of such links. However, a hypothesis may reside in bacterial modulatory ability as suggested by Alcock and colleagues [58], who described a potential involvement of microbes in the manipulation of eating behavior by altering the host preferences through a modulation of receptor expression, as in vivo animal model studies on gut microbiota showed [59,60]. Another plausible explanation may lie in bacterial ability to degrade carbohydrates into disaccharides, monosaccharides, and organic acids, used as “building material” for biofilms [61]. The physical barrier between tastants and taste receptors would, as a consequence, be less or more efficient, thus influencing sensitivity. According to our results, also Feng and colleagues [62] reported that a higher proportion of *Actinobacteria* was linked to lower taste sensitivity, while a higher proportion of *Bacteroidetes* increased sensitivity. 

Nonetheless, a more detailed characterization of microbial communities and their metabolic feature would be of interest, but this study supports that the oral microbiota composition deserves to be considered as an influencing variable when investigating peri-receptor events involved in chemosensory processes. 

The role of diet in shaping the gut microbiota is widely recognized [63,64]. However, until recently, only a few studies have considered the association between habitual diet and oral microbiota. 

In the present study, interesting correlations between the relative abundance of bacterial taxa on tongue dorsum and dietary intake were found. Indeed, *Clostridia* class was positively associated with total energy, fat, and protein intake but negatively associated with fiber intake, whereas *Proteobacteria* phylum and *Prevotella* genus showed the opposite association. Since it has been found that oral cavity and stool bacteria overlapped in nearly half (45%) of the subjects in recent studies [65,66], it is possible to hypothesize that dietary habits could affect both oral and gut microbiota in a similar way. Indeed, our results are in line with the general assumption that some gut microbial taxa are positively associated with vegetable-rich (*Prevotella*) or protein/fat-rich diets (*Clostridia*) [67,68]. However, further studies are warranted to clarify whether observations from the gut microbiome are transferrable to the oral microbiome. In this context, the oral microbiome could be further investigated as potential marker of long-term consumption of healthy or unhealthy diets.

The strengths of the present study include an investigation of the relationship between taste sensitivity for all the four tastes with a range of parameters of food consumption frequency and food intake. In particular, dietary intake was investigated through assessment of actual food intakes (seven-day food records) and habitual intakes of different categories of foods and beverages (FB-FQ), capturing different aspects of eating habits. Finally, the multidisciplinary approach applied in the present study offers new insights into the reciprocal impact between taste perception, food intake, and oral microbiota composition.

The present study has also several limitations. Firstly, participants involved were a small sample of Italian women and men of similar age (young) and BMI (normal range). Therefore, the findings of this study cannot be generalized to other ethnicities, ages, or BMI groups. Secondly, the study design was cross-sectional and the findings represent only relationships among variables under study while no causations can be ascertained. Thirdly, limitations to the study include validity of food intake measurements. Reported intakes may be inaccurate due to memory recall, interviewer and subject bias, and responder fatigue, all of which contribute to underestimating or overestimating food intake measures [69].

In conclusion, the present study shows a link between taste sensitivity and dietary measurements in a group of young healthy women and men with normal BMI and food intake. Moreover, significant relationships between taste sensitivity and dietary measurements, but also with oral microbiota composition, were found.

These findings have implications for eating behavior, as perceived sensory properties of foods and beverages clearly influence preferences and the type and amount of food consumed [1]. Moreover, this study provides further support that nongenetic factors, such as the oral bacteria lining the tongue, should be adequately considered in order to gain new insights into taste-related eating habits that may influence long-term health outcomes. The impact of genetic and nongenetic characteristics, including the complex interactions among multiple factors related with food cues and exposure, can affect food choices and dietary intake. For this reason, this topic remains an important research area to be further investigated, since all these aspects reciprocally influence each other, driving towards individual eating behavior.

## Figures and Tables

**Table 1:** Concentrations (g/L) of sucrose, sodium chloride, caffeine, and citric acid used to determine recognition thresholds.

Taste Quality	Reference Stimuli	Sample Concentration (g/L) ^a^						
		1	2	3	4	5	6	7
Sweet	Sucrose	1.6 × 10^−1^	4.0 × 10^−1^	1.02	2.56	6.4	16.0	40.0
Salty	Sodium chloride	6.25 × 10^−2^	1.25 × 10^−2^	2.5 × 10^−1^	5.0 × 10^−1^	1.0	2.0	4.0
Bitter	Caffeine	3.0 × 10^−3^	9.0 × 10^−3^	3.0 × 10^−2^	8.0 × 10^−2^	2.4 × 10^−1^	8.0 × 10^−1^	2.0
Sour	Citric acid	2.0 × 10^−2^	5.0 × 10^−2^	8.0 × 10^−2^	1.5 × 10^−1^	3.5 × 10^−1^	7.5 × 10^−1^	1.5

^a^ The concentration series for sucrose, sodium chloride, and caffeine were prepared with successive 0.4 log dilution steps. The concentration series for citric acid were prepared with successive 0.3 log dilution steps. Reference chemical details: sucrose (Sigma Aldrich srl, Milano, Italy), sodium chloride (Sigma Aldrich srl, Milano, Italy), caffeine (Sigma Aldrich srl, Milano, Italy), and citric acid (Sigma Aldrich srl, Milano, Italy).

**Table 2:** The six frequency response options and their conversion into Daily Equivalent Frequency.

Original Frequency used in FFQ	Daily Equivalent Frequency
Less than once per month	0.02
1–3 times per month	0.07
1–4 times per week	0.43
5–7 times per week	0.86
2–4 times per day	3.00
5 or more times per day	5.00

FFQ: Food Frequency Questionnaire.

**Table 3:** Baseline characteristics, energy and nutrient intake, and gustatory functions (taste thresholds, PROP responsiveness, and FPD) presented as mean, standard error of study participants.

	Mean	SEM
Age (years)	23.3	0.3
BMI (kg/m^2^)	21.6	0.3
*Gustatory Functions*		
Sweet threshold (g/L)	3.61	0.62
Salty threshold (g/L)	0.20	0.02
Bitter threshold (g/L)	0.16	0.04
Sour threshold (g/L)	0.09	0.01
*Food Intake*		
Total Energy (kcal)	1829	60
Protein (%) ^a^	15.6	0.4
Fat (%) ^a^	35.2	0.9
Carbohydrates (%) ^a^	45.2	0.7
Protein (g) ^b^	68.0	2.3
Fat (g) ^b^	70.1	2.7
Carbohydrates (g) ^b^	216.0	8.6
Total Fiber (g) ^b^	15.1	6.7

^a^ Calculated as% of total energy intake (kcal); ^b^ Calculated as gram per day.

**Table 4:** Mean values of daily equivalent frequency consumption for the 22 food categories by salty and sweet taste sensitivity level.

Items	Daily Equivalent Frequency				Daily Equivalent Frequency			
	*p value*	Salty Taste Sensitivity Level			*p value*	Sweet Taste Sensitivity Level		
		Hyper	Normal	Hypo		Hyper	Normal	Hypo
Cereal and cereal-derived products (e.g., pasta, rice, barley, spelt)	0.07	1.67	1.33	2.23	0.88	1.92	1.58	1.49
Salty baked products (e.g., bread, pizza, focaccia)	**0.007**	0.99 ^b^	1.45 ^ab^	1.99 ^a^	0.69	1.37	1.12	1.40
Bakery products (e.g., bakery and breakfast cereals, biscuits, croissants)	**0.04**	0.76 ^b^	1.39 ^a^	1.42 ^a^	0.48	1.12	1.34	0.94
Meats	0.98	0.50	0.51	0.49	0.85	0.48	0.54	0.50
Cured meats	0.64	0.36	0.40	0.31	0.09	0.27	0.48	0.37
Fish	0.16	0.41	0.28	0.43	0.35	0.39	0.28	0.39
Milk and yoghurts	0.47	0.84	0.75	1.11	0.27	0.75	0.61	1.04
Dairy products	0.32	0.51	0.79	0.79	0.19	0.52	0.62	0.86
Eggs	0.22	0.34	0.23	0.29	0.72	0.33	0.27	0.30
Vegetables	0.41	1.80	1.85	2.40	*0.08*	2.62	1.72	1.71
Legumes	**0.05**	0.36 ^b^	0.67^a^	0.41 ^ab^	**0.03**	0.69 ^a^	0.41 ^ab^	0.35 ^b^
Potatoes	0.20	0.35	0.58	0.47	0.65	0.49	0.35	0.41
Fruit	0.22	1.65	1.84	2.42	0.79	2.07	1.78	1.78
Fruit juices	0.31	0.22	0.43	0.48	0.80	0.40	0.26	0.36
Nuts	0.27	0.23	0.45	0.36	0.76	0.33	0.22	0.33
Sweets and desserts (e.g., cakes, ice creams, chocolate)	0.53	0.60	0.86	0.78	**0.02**	0.32 ^b^	0.98 ^a^	0.87 ^a^
Fats	**0.05**	0.26 ^b^	0.22 ^b^	0.45 ^a^	0.18	0.19	0.35	0.34
Oils	0.47	1.99	1.54	1.72	0.87	1.83	1.94	1.74
Salty snacks (e.g., chips, salty peanuts)	0.23	0.22	0.22	0.35	0.23	0.17	0.27	0.30
Alcoholic beverages	0.21	0.49	0.29	0.31	0.18	0.30	0.56	0.35
Soft drinks	**0.007**	0.26 ^b^	1.13 ^a^	0.16 ^b^	0.82	0.59	0.39	0.38
Candies and gums	0.82	0.63	0.54	0.78	0.32	0.82	0.81	0.40

Significant *p*-values are shown in bold. Different letters indicate significant differences according to Bonferroni’s post hoc test.

**Table 5:** Mean values of daily equivalent frequency consumption for the 22 food categories by bitter and sour taste sensitivity level.

Items	Daily Equivalent Frequency				Daily Equivalent Frequency			
	*p value*	Bitter Taste Sensitivity Level			*p value*	Sour Taste Sensitivity Level		
		Hyper	Normal	Hypo		Hyper	Normal	Hypo
Cereal and cereal-derived products (e.g., pasta, rice, barley, spelt)	0.58	1.75	1.29	1.66	0.08	2.19	1.31	1.78
Salty baked products (e.g., bread, pizza, focaccia)	0.70	1.45	1.25	1.20	0.11	0.90	1.43	1.66
Bakery products (e.g., bakery and breakfast cereals, biscuits, croissants)	0.81	0.92	1.10	1.10	0.21	0.69	1.20	1.29
Meats	0.65	0.47	0.48	0.54	0.75	0.53	0.51	0.46
Cured meats	0.41	0.34	0.46	0.33	0.37	0.35	0.40	0.29
Fish	0.22	0.42	0.26	0.36	**0.004**	0.49 ^a^	0.27 ^b^	0.46 ^a^
Milk and yoghurt	0.66	0.72	0.97	0.92	0.96	0.91	0.84	0.82
Dairy products	0.07	0.52	0.52	0.95	0.55	0.49	0.75	0.69
Eggs	0.52	0.33	0.24	0.30	0.54	0.36	0.31	0.27
Vegetables	0.08	1.92	1.04	2.23	0.33	1.84	1.77	2.39
Legumes	0.51	0.53	0.42	0.38	0.09	0.32	0.61	0.40
Potatoes	0.53	0.49	0.39	0.35	0.23	0.61	0.42	0.36
Fruit	0.76	2.06	1.70	1.81	0.94	1.77	1.94	1.94
Fruit juices	0.24	0.48	0.39	0.19	0.70	0.22	0.31	0.39
Nuts	0.42	0.43	0.26	0.26	0.41	0.43	0.23	0.36
Sweets and desserts (e.g., cakes, ice creams, chocolate)	0.85	0.73	0.61	0.78	0.44	0.46	0.75	0.79
Fats	0.84	0.29	0.29	0.25	0.26	0.26	0.36	0.22
Oils	**0.007**	1.46 ^b^	1.11 ^b^	2.22 ^a^	0.35	1.64	1.72	2.16
Salty snacks (e.g., chips, salty peanuts)	0.40	0.24	0.20	0.32	0.44	0.18	0.28	0.22
Alcoholic beverages	0.34	0.46	0.33	0.29	0.70	0.31	0.43	0.37
Soft drinks	0.22	0.68	0.20	0.16	0.81	0.40	0.56	0.36
Candies and gums	0.91	0.68	0.73	0.57	0.76	0.48	0.63	0.78

Significant *p*-values are shown in bold. Different letters indicate significant differences according to Bonferroni’s post hoc test.
